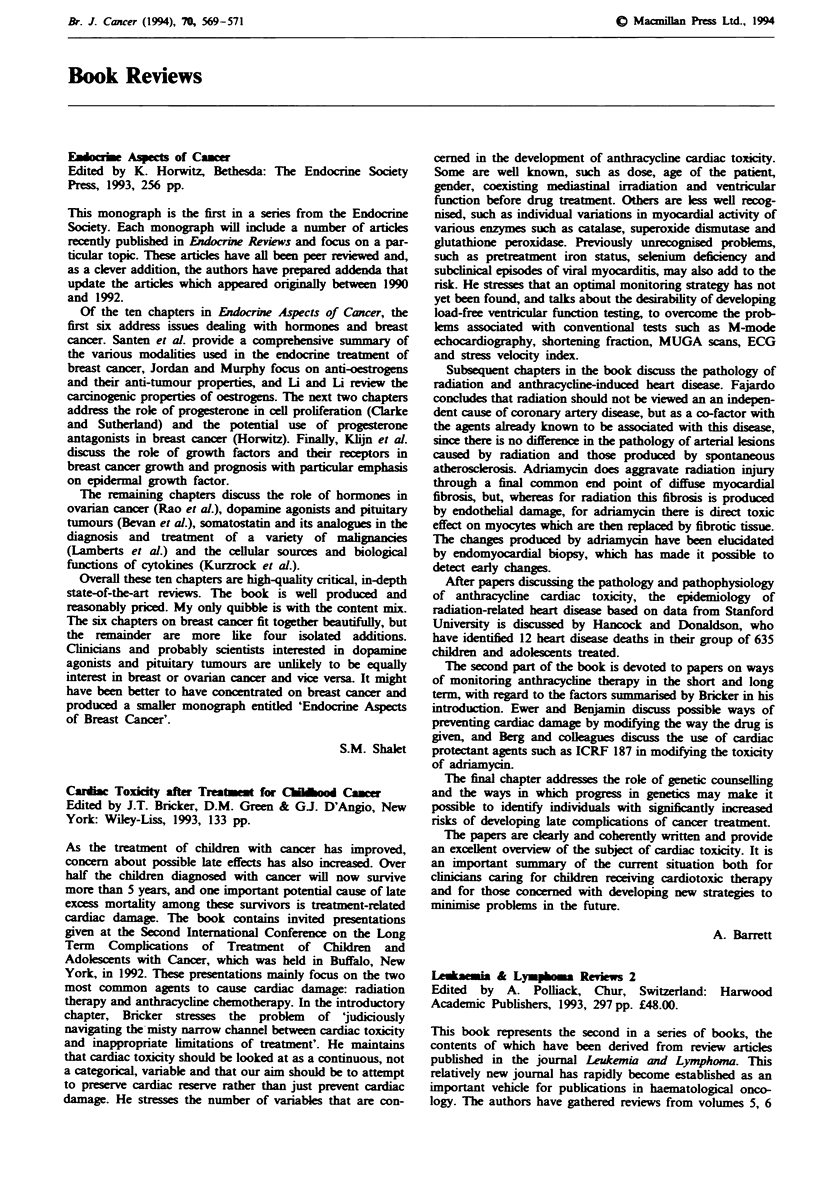# Cardiac toxicity after treatment for childhood cancer

**Published:** 1994-09

**Authors:** A. Barrett


**Cardiac Toxicity after Treatment for Childhood Cancer**

Edited by J.T. Bricker, D.M. Green & G.J. D'Angio, New York: Wiley-Liss, 1993, 133 pp.

As the treatment of children with cancer has improved, concern about possible late effects has also increased. Over half the children diagnosed with cancer will now survive more than 5 years, and one important potential cause of late excess mortality among these survivors is treatment-related cardiac damage. The book contains invited presentations given at the Second International Conference on the Long Term Complications of Treatment of Children and Adolescents with Cancer, which was held in Buffalo, New York, in 1992. These presentations mainly focus on the two most common agents to cause cardiac damage: radiation therapy and anthracycline chemotherapy. In the introductory chapter, Bricker stresses the problem of 'judiciously navigating the misty narrow channel between cardiac toxicity and inappropriate limitations of treatment'. He maintains that cardiac toxicity should be looked at as a continuous, not a categorical, variable and that our aim should be to attempt to preserve cardiac reserve rather than just prevent cardiac damage. He stresses the number of variables that are concerned in the development of anthracycline cardiac toxicity. Some are well known, such as dose, age of the patient, gender, coexisting mediastinal irradiation and ventricular function before drug treatment. Others are less well recognised, such as individual variations in myocardial activity of various enzymes such as catalase, superoxide dismutase and glutathione peroxidase. Previously unrecognised problems, such as pretreatment iron status, selenium deficiency and subclinical episodes of viral myocarditis, may also add to the risk. He stresses that an optimal monitoring strategy has not yet been found, and talks about the desirability of developing load-free ventricular function testing, to overcome the problems associated with conventional tests such as M-mode echocardiography, shortening fraction, MUGA scans, ECG and stress velocity index.

Subsequent chapters in the book discuss the pathology of radiation and anthracycline-induced heart disease. Fajardo concludes that radiation should not be viewed an an independent cause of coronary artery disease, but as a co-factor with the agents already known to be associated with this disease, since there is no difference in the pathology of arterial lesions caused by radiation and those produced by spontaneous atherosclerosis. Adriamycin does aggravate radiation injury through a final common end point of diffuse myocardial fibrosis, but, whereas for radiation this fibrosis is produced by endothelial damage, for adriamycin there is direct toxic effect on myocytes which are then replaced by fibrotic tissue. The changes produced by adriamycin have been elucidated by endomyocardial biopsy, which has made it possible to detect early changes.

After papers discussing the pathology and pathophysiology of anthracycline cardiac toxicity, the epidemiology of radiation-related heart disease based on data from Stanford University is discussed by Hancock and Donaldson, who have identified 12 heart disease deaths in their group of 635 children and adolescents treated.

The second part of the book is devoted to papers on ways of monitoring anthracycline therapy in the short and long term, with regard to the factors summarised by Bricker in his introduction. Ewer and Benjamin discuss possible ways of preventing cardiac damage by modifying the way the drug is given, and Berg and colleagues discuss the use of cardiac protectant agents such as ICRF 187 in modifying the toxicity of adriamycin.

The final chapter addresses the role of genetic counselling and the ways in which progress in genetics may make it possible to identify individuals with significantly increased risks of developing late complications of cancer treatment.

The papers are clearly and coherently written and provide an excellent overview of the subject of cardiac toxicity. It is an important summary of the current situation both for clinicians caring for children receiving cardiotoxic therapy and for those concerned with developing new strategies to minimise problems in the future.

A. Barrett